# Development and application of artificial intelligence in traditional Chinese medicine research and development

**DOI:** 10.1186/s13020-025-01288-7

**Published:** 2026-01-08

**Authors:** Anxin Wang, Qiaoxian Luo, Xiaotian Tan, Yixin Yao, Xuebo Peng, Hua Luo, Yuanjia Hu

**Affiliations:** 1 State Key Laboratory of Mechanism and Quality of Chinese Medicine, Institute of Chinese Medical Sciences, University of Macau, Macao SAR, China; 2 The Centre for Pharmaceutical Regulatory Sciences, University of Macau, Macao SAR, China; 3 Guangdong Foshan Joint Key Laboratory for the Research and Development and Industrialization of Chinese Medicinal Formulations, Foshan, 528000 China; 4 Guangdong Provincial Hospital of Integrated Traditional Chinese and Western Medicine, Foshan, 528000 China; 5 Guangxi University of Chinese Medicine, Nanning, 530001 China

**Keywords:** AI, TCM R&D, Drug design, Machine learning, Deep learning

## Abstract

**Background:**

The integration of artificial intelligence (AI) into traditional Chinese medicine (TCM) research and development offers promising solutions to longstanding challenges in the field. These challenges include the complexity of TCM formulations, variability in quality control, and hurdles in global market acceptance. The unique synergy between AI technologies and TCM principles creates opportunities to enhance research efficiency, standardization, and innovation.

**Aim of review:**

This review aims to explore the applications and impact of AI across three critical stages of TCM development: drug design, pharmaceutical manufacturing, and market access. By summarizing the advancements and limitations in these areas, the review identifies the transformative potential of AI and proposes future directions for integrating AI with emerging technologies to advance TCM research and development (R&D).

**Key scientific concepts of review:**

AI has transformative potential in TCM development, addressing key challenges across various stages. In drug design, AI accelerates the identification of active compounds, optimizes formula composition, and models pharmacodynamic relationships to enhance innovation efficiency and precision. During pharmaceutical manufacturing, AI contributes to process optimization, quality control, and the standardization of TCM products, ensuring stable and scalable production. For market access, although no TCM developed by AI has entered the clinic, AI has played a role in comprehensive safety and efficacy assessments and simplified regulatory compliance in other drugs. By leveraging these advances and reviewing limitations, AI promotes the need to develop more integrated, more efficient,

and more utilized methods in TCM R&D.

## Introduction

As an important part of traditional Chinese culture, TCM has a history of thousands of years and has gained rich clinical experience in treating and preventing diseases. However, with the development of modern medicine, TCM faces many challenges in global promotion and modernization research, such as the lack of a standardized drug quality evaluation system, long R&D cycle, and unclear drug mechanisms. Especially in the process of new drug development, due to the complex drug formula and diverse ingredients, the research of TCM requires a lot of experimental verification and clinical data support, resulting in low efficiency of new drug development and high resource consumption.

In recent years, AI technology has risen rapidly and shown great potential in healthcare. With its powerful data processing and analysis capabilities, many AI models for TCM have emerged (Table 1). Especially in the field of TCM R&D, AI can mine potential drug ingredients, screen active compounds, and predict drug targets in massive amounts of data, greatly improving the accuracy of drug screening and development [1, 2]. For example, TCMBank has developed a network that uses a variety of algorithms to display the complex relationship between Chinese herbal medicines, ingredients, potential targets, and diseases, effectively improving the efficiency of TCM research and development [3]. The INPUT database designed by Li et al. includes 4,716 herbs, 29,812 herbal compounds, and 9,847 diseases, which can help new discoveries of TCM through the “herb-compound-gene-disease network” [4].

**Table 1:** Overview of AI-based models for TCM development

Model Name	Developer/Organization	Core Technology	Focus Area in TCM
Digital Herbalism	Tianshili, Huawei	Pre-training of massive TCM text data, fine-tuning for multiple scenarios of TCM R&D	Discovery of natural medicines, production of new formulas
Herbal Intelligence Library	Chengdu University of Traditional Chinese Medicine, Baidu, Taiji Group, Tianfu TCM City	Knowledge graph of the whole industry chain of TCM	Extraction and generation of TCM knowledge, output of vertical solutions, one-stop digital services for the TCM industry
Jiu Wei-TCM-LLM	Jiuwei Health, Huawei Cloud	Knowledge base of TCM, feature extraction and contextual understanding capabilities	Drug analysis, ADME/t evaluation, target prediction, network analysis, drug combination analysis
ChatDD	Boao Jingfang, Shuimu Molecule	Multimodal data fusion and understandin	Services for the whole process of pharmaceutical R&D, talent cultivation
Hua Tuo-TCM-LLM	Bozhou Municipal Government, Huawei	Building a comprehensive knowledge base of TCM	TCM industry transaction platforms, medicinal material traceability
Qihuang Wendao-TCM-LLM	Dajing Traditional Chinese Medicine	Knowledge graph of TCM diagnosis and treatment	Clinical diagnosis and treatment, TCM and health care regulation programmes
‘Guwa’Intelligent Medical Assistant	Baidu, Gushengtang	Integration of clinical e-records of masters of national medicine, case information	Intelligent diagnosis and guidance, assisted diagnosis and treatment
Xunfei Xinghuo-TCM-LLM	iFLYTEK	In-depth understanding of patients' linguistic expressions and needs and precise responses	Assisted diagnosis and treatment, health consultation
Haihe Qibao-LLM	TianDa ZhiTu, Tianjin University of Traditional Chinese Medicine, Haihe Laboratory of Modern Traditional Chinese Medicine	Constructing a high-quality knowledge graph of TCM	Intelligent diagnosis and treatment, electronic case generation [190]
Bian Cang-TCM-LLM	QiLu University of Technology, Affiliated Hospital of Shandong University of Traditional Chinese Medicine	Medical data from the hospital affiliated to Shandong University of Traditional Chinese Medicine	Assisted diagnosis and treatment in TCM, case information management [191]
Heng Qin-TCM-LLM	Hengqin Laboratory	10 billion characters of TCM knowledge text and digitised cases from TCM hospitals	Assisted diagnosis and treatment in TCM
Zhong Jing-TCM-LLM	Fudan University, Tongji University	Rigorous Prompt templates	Reasoning on TCM prescription data and diagnostic thinking logic [192]
Shen Nong-TCM	East China Normal University	Using entity-centred self-instruction approach, 26,000 + TCM instruction datasets	Medical consultation [193]
Xuanqi Wendui- LLM	Zhejiang University of Traditional Chinese Medicine, Dr. GanCao	Converged Knowledge Graphs	Case information management, assisted diagnosis and treatment
Tianhe Lingjiu- LLM	National Supercomputer Tianjin Centre, Tianjin University of Traditional Chinese Medicine	Library of TCM masterpieces and clinical evidence-based evidence on acupuncture	Personalised recommendation of acupuncture treatments
Shuzhi Qihuang -TCM-LLM	East China Normal University, Shanghai University of Traditional Chinese Medicine	Knowledge graph for high quality TCM	Intelligent quiz on knowledge of TCM, health consultation, dynamic knowledge graph on TCM Interaction [194]
TCMLLM	Beijing Jiaotong University	Created Prescription recommendation instruction fine-tuning dataset with 68,000 data entries	TCM assisted diagnosis and treatment, TCM knowledge Q&A
Huang-Di-LLM	Nanjing University, Zhengzhou University	Incorporating rich TCM textbook data	TCM antiquity knowledge Q&A
APUS Zhicao-LLM	APUS	Aligned with the experience of TCM veterans through fine-tuning and RLHF training techniques	Medicine prescription knowledge consulting, toxic TCM consulting, TCM prescription recommendation, TCM medication recommendation
Naoqi Suwen-LLM	Nanhu Research Institute	Brain-like transformation using dendritic neuron dynamics model	Precision diagnosis support
Ben Cao-LLM	Harbin Institute of Technology, Health Intelligence Group of Information Retrieval Research Centre	Large language model set for Chinese medical instruction fine-tuning/instruction fine-tuning	TCM assisted diagnosis and treatment [195]
Simiao Sun Chinese Medical-LLM	East China University of Science and Technology	100,000 high-quality Chinese medical data	TCM assisted diagnosis and treatment
Qi Zhen-GPT	Zhejiang University	Chinese Medical Instruction Dataset constructed by Qizhen Medical Knowledge Base Instruction dataset	Medicine knowledge Q&A
TCM-GPT	Beijing University of Posts and Telecommunications	Large corpus of TCM	TCM assisted diagnosis and treatment [196]
MedChatZH	East China University of Science and Technology	Fine-tuning with medical instruction datasets	TCM QA dialogue model [197]
TCM-FTP	The Hong Kong University of Science and Technology	Medical records of senior specialists in digestive system diseases	TCM prescription prediction [198]
CPMI-Chat GLM	Anhui University of Traditional Chinese Medicine	Chinese patent medicine instructions (CPMI) dataset	TCM medication recommendation [199]
Shennong Alpha	Westlake University	Multi-dimensional knowledge graph of medicinal materials, ingredients, targets and diseases	Standardized description and access to key information of natural medicinal materials [200]
TCMChat	Innovation Center of Yangtze River Delat,Zhe jiang University	Constructed over 1 Gb of unsupervised data and about 600,000 pairs of supervised instruction data	TCM or prescription recommendation and ADMET property prediction [201]

**Fig. 1:**
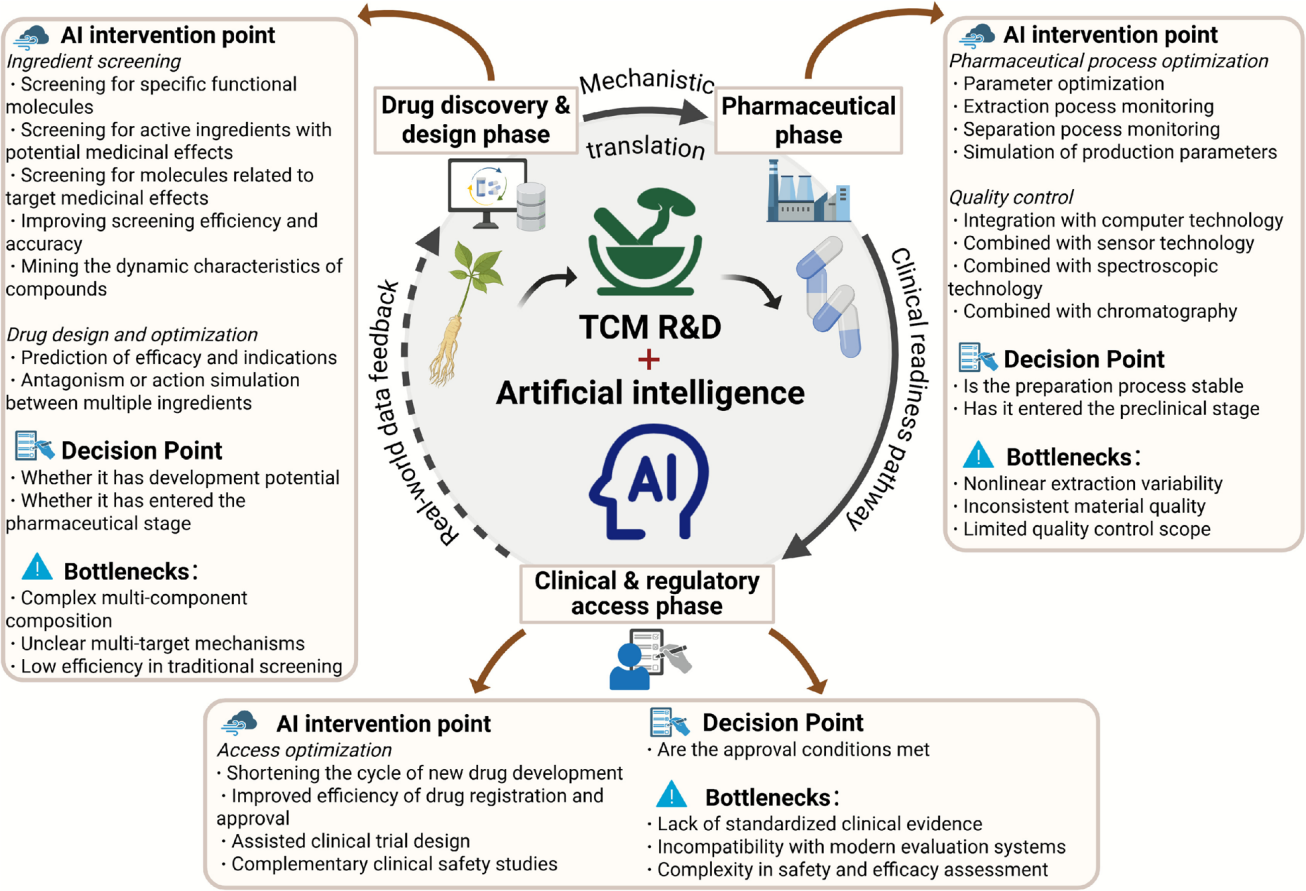
The application of AI in key phases of TCM research and development, including drug discovery and design phase, pharmaceutical processing phase and clinical and regulatory access phase. At each phase, AI assists in ingredient screening, process optimization, trial design, and decision-making, while addressing key bottlenecks and incorporating real-world data feedback

The research and development of TCM mainly consists of three stages: the design stage of new TCM drugs, the pharmaceutical stage of new TCM drugs, and the pre-market stage of new TCM drugs. TCM is known for its complex composition, exquisite compatibility, and diverse mechanisms of action. However, this characteristic also poses huge challenges to its research and development, such as difficulties in screening active ingredients, long formulation design cycles, and time-consuming and labor-intensive research on pharmacological mechanisms. The introduction of AI provides a new way of thinking to solve these problems. Using big data analysis and machine learning techniques, AI can quickly screen for potential active ingredients, predict their pharmacological properties, and optimize formulation ratios, thereby significantly shortening the research and development cycle [5]. In addition, AI also plays an indispensable role in efficacy research, which can greatly improve research efficiency by constructing efficacy models and simulating experiment results, promoting the development of TCM towards a more scientific and efficient direction. These unique values demonstrate the important role of AI in promoting TCM innovation (Fig. 1).

In recent decades, the TCM and computer science communities have generated a large number of theories and methods for the R&D of new TCM drugs, but these efforts have not been systematically organized and summarized. Therefore, this paper attempts to comprehensively review the three key stages of AI in the field of TCM research and development from the perspective of the entire new drug R&D. This will promote the deeper application of AI in the field of TCM and better promote the development and transformation of new TCM drugs.

### Development and application of AI in the drug design stage of TCM

#### Challenges in the drug design stage

The design stage of TCM has distinct characteristics, including ingredient screening, pharmacodynamic mechanism exploration, pharmacological ingredient optimization, compound optimization, quantitative structure–activity relationship (QSAR) modeling, and de novo drug design. Ingredient screening requires screening out core active ingredients with potential therapeutic effects from the complex chemical components of TCM; pharmacodynamic mechanism exploration focuses on revealing the drug's targets and signaling pathways; pharmacological ingredient optimization aims to improve efficacy and reduce toxic side effects through structural modification or compatibility adjustment; compound optimization is divided into ligand-based compound optimization and structure-based compound optimization; QSAR modeling focuses on predicting the pharmacological properties of compounds based on their chemical structure; de ​​novo drug design mainly creates new molecules based on the desired properties. For example, high-throughput screening techniques and isolation and identification methods, such as HPLC and LC–MS-based studies, have shown that tanshinone compounds have a significant impact on cardiovascular diseases. [6]. Network pharmacology has been used to analyze the regulatory effect of curcumin on the PI3K-Akt signaling pathway [7]. Through structural modification strategies, such as co-crystal engineering to improve the physicochemical and biopharmaceutical properties of active pharmaceutical ingredients (T-APIs) extracted from TCM to improve bioavailability, or adjust the compatibility ratio of compound prescriptions, such as optimizing the effect of a certain TCM compound in the treatment of arthritis to improve efficacy [8].

However, this stage faces many key pain points. TCM ingredients have complex structures and are mostly mixtures, making it difficult to accurately analyze their chemical properties and biological activities. In addition, the compatibility of TCM involves the synergistic effects of multiple ingredients and multiple targets, and traditional experimental methods make it difficult to fully analyze their mechanisms of action [9, 10]. For example, the basic research of TCM is based on its unique properties, such as medicinal properties, odors, and channels, and the interactions between drugs form the theoretical basis of compatibility, including synergistic and antagonistic effects [11]. On the one hand, external compatibility needs to consider the qualitative and quantitative changes of multiple drug ingredients, which is extremely complex. On the other hand, internal compatibility involves the precise analysis of the absorption, metabolism and synergistic effects of drug ingredients in the body, especially the mechanism of action of multiple targets and pathways, which is difficult to fully quantify and verify [12].

These challenges urgently need to be combined with modern technical means, such as high-throughput screening [13], network pharmacology [14] and genome sequencin [15], to accelerate the research and development of TCM drugs.

#### Application of AI in ingredient screening and analysis

**Fig. 2:**
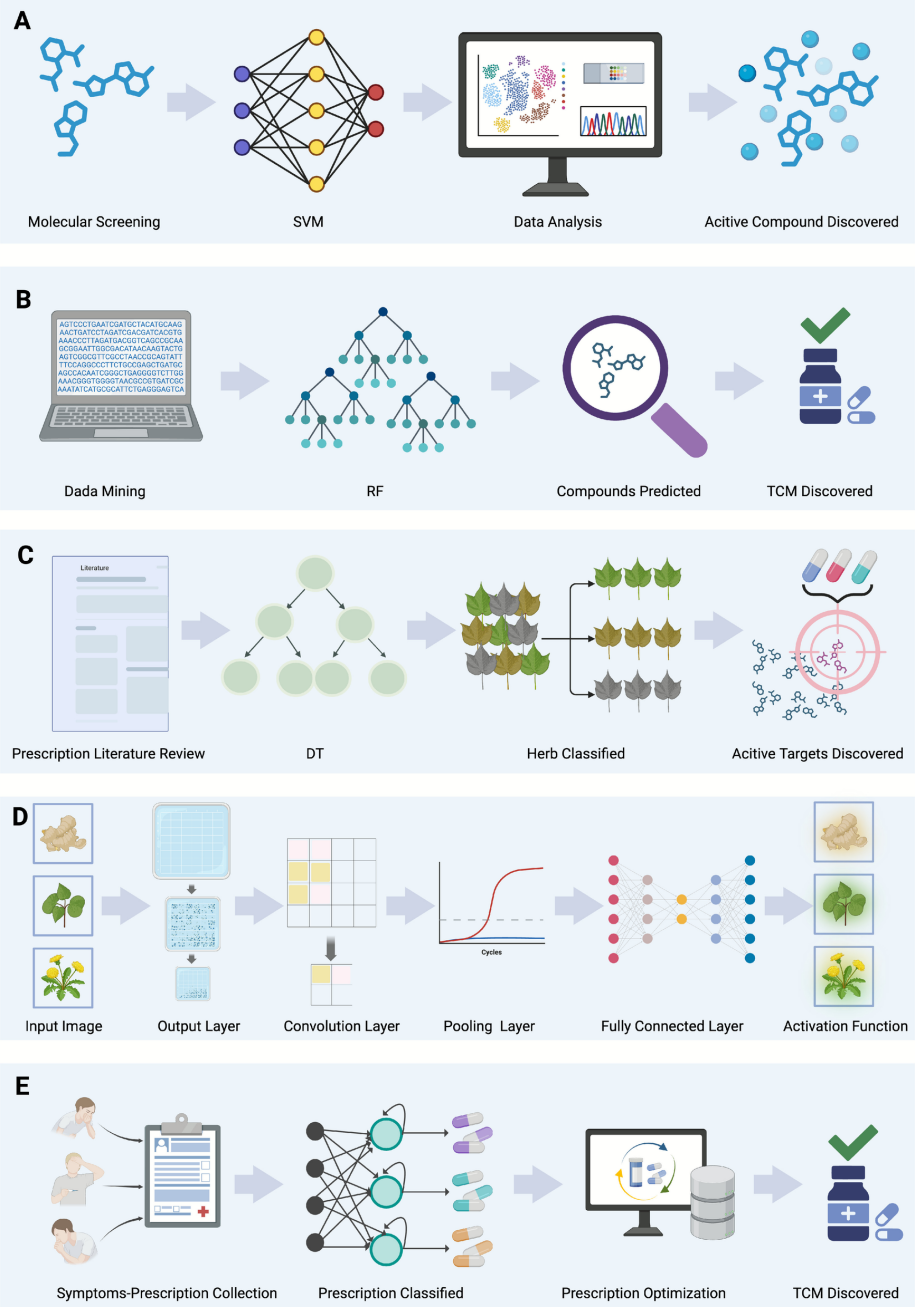
Applications of five key algorithms in the design stage of TCM. **A** Application of the SVM algorithm to screen effective molecular components of TCM; **B** Use of the RF algorithm to identify TCM compounds, revealing herbal medicines with potential active ingredients; **C** Deployment of the DT algorithm to extract targets from literature data, identifying associations between TCM and therapeutic effects, and enabling classification; **D** Utilization of CNN to recognize structural and chemical features of TCM, supporting the evaluation of effective medicinal materials and optimization of drug design; **E** Implementation of the RNN algorithm to capture relationships between symptoms and TCM prescriptions, facilitating the optimization of herbal formulations

The screening of active ingredients in TCM faces huge challenges due to the complexity of its ingredients. Traditional methods are not only time-consuming and inefficient, but also hinder clinical translation and commercial application. In recent years, in particular, AI technologies such as machine learning (and deep learning) have brought breakthroughs in ingredient screening by optimizing experimental design, reducing unnecessary experimental screening processes [16, 17]. Generally speaking, common AI practices include machine learning (ML) and deep learning (DL), which is a part of machine learning [18, 19]. These two AI practices have been widely explored by scientists, and 15 common algorithmic models have emerged: linear regression (LR) [20, 21], logistic regression [22, 23], decision trees (DT) [24, 25], random forests (RF) [26, 27], support vector machines (SVM) [28–30], k-nearest neighbors (KNN) [31], principal component analysis (PCA) [32], naive Bayes [33, 34], AdaBoost [35, 36], hidden Markov models (HMMs) [37], recurrent neural network (RNN) [38, 39], convolutional neural network (CNN) [40–42], t-distributed Stochastic Neighbor Embedding (t-SNE) [43, 44], generative adversarial network [45, 46], reinforcement learning (RL) [47](Table 2). For example, Shang and Zhao used a combination of multiple models (including SVM, KNN, NB, DT, RF, AdaBoost, and LR) to construct a model for screening hepatotoxic compounds, which can effectively screen hepatotoxic compounds in traditional Chinese-Western medicine [21]. Such practices show that, since TCM research emphasizes the overall mechanism and multi-target synergy, it often involves the integration of complex component combinations and traditional experience, while conventional drug development focuses on the precise effects of single components and targets. Therefore, at present, "AI + TCM" research usually needs to integrate multiple algorithms (such as SVM, RF, DT, CNN, RNN) to explore from multiple perspectives (Fig. 2) in order to take into account both the overall nature and the specificity.

**Table 2:** The characteristic and application of 15 various algorithms in TCM screening

Algorithm	Characteristic	Data requirements	Application	Computational cost
Linear Regression	Models linear relationships between variables by minimizing the sum of squared errors. Suitable for predicting continuous variables	Moderate datasets (≥ 100), assumes linearity and low multicollinearity	Analyzing the relationship between the concentration of TCM components and their therapeutic effects, such as the contribution of a single compound to a specific efficacy [21]	Low, fast training and inference
Logistic Regression	A classification algorithm for binary outcomes that outputs probabilities using a logistic function	Moderate datasets (≥ 100–200); performs well with linearly separable features	Determining whether a TCM formulation has specific effects (e.g., anti-inflammatory) or toxicity risks (e.g., hepatotoxicity) [21]	Low, computationally efficient
DT	A rule-based model represented as a tree structure, simple and interpretable, but prone to overfitting	Works with small to medium datasets (≥ 200); accepts mixed-type features	Screening TCM ingredient combinations and deriving classification rules for their effects or toxicities [21, 54, 55]	Low–Moderate, very fast inference
RF	An ensemble of decision trees, improving model accuracy and robustness, and handling high-dimensional data effectively	Larger datasets (≥ 500); performs well even with noisy or incomplete data	Identifying key active ingredients in TCM formulations and assessing their contributions to therapeutic effects or toxicities [21, 48, 51–54]	Moderate, where m is number of trees. Parallelizable
SVM	Finds a hyperplane for classification or regression, performs well in high-dimensional spaces and with small datasets	Small to medium datasets (100–500); feature scaling needed	Classifying whether a TCM component has specific pharmacological activity or predicting toxicity [21, 48–50]	Moderate–High, not ideal for large datasets
KNN	A distance-based classification or regression algorithm, simple to implement but computationally intensive	Requires normalized data; suitable for ≤ 1000 samples	Predicting the likely functions of unknown TCM components based on similarity to known active ingredients [21, 54]	High at inference, slow with large datasets
PCA	A dimensionality reduction method that extracts the main features of data while preserving key information	Works best when number of features > samples (p > n)	Reducing the complexity of multi-component TCM formulations to identify the most influential components [202]	Low–Moderate, typically fast
NB	A probabilistic classifier based on Bayes’ theorem, assuming feature independence	Small datasets (≥ 100); assumes conditional independence	Quickly evaluating the likelihood of TCM ingredient combinations contributing to specific therapeutic effects [21, 54]	Low, highly efficient
AdaBoost	An ensemble learning technique that combines multiple weak classifiers into a strong classifier	Requires diverse training samples; sensitive to outliers	Improving classification accuracy in identifying effective TCM component combinations [21, 54]	Moderate
HMMs	Probabilistic models for sequential data, capturing relationships between hidden states and observed data	Sequence data with labeled states; ≥ 100 sequences	Analyzing metabolic pathways of TCM ingredients in the body or predicting dynamic changes in therapeutic effects	Moderate
RNN	A neural network designed for sequence data, capable of capturing long-term dependencies	Requires many labeled sequences; sequence length-sensitive	Modeling time-dependent relationships between TCM components and physiological indicators [61–63]	High, training time grows with sequence length; needs GPU
CNN	Excels at processing image and structured data, extracting local features through convolution operations	Image datasets ≥ 1000; consistent input shapes	Recognizing the chemical structure of TCM components or analyzing TCM microscopy images [56–59]	High, requires GPUs; complex architectures increase cost
t-SNE	A nonlinear dimensionality reduction technique for data visualization, preserving local structure	Up to 5000 samples due to O(n^2^) complexity	Visualizing complex datasets, such as clustering TCM components or samples based on their features [203]	Moderate–High, suited for small to medium datasets
GAN	Composed of a generator and a discriminator, capable of generating realistic synthetic data	Requires large datasets and careful tuning; prone to instability	Generating new TCM molecular structures and predicting their potential therapeutic effects or toxicity	Very High, requires GPU and extensive tuning
RL	A trial-and-error-based approach that optimizes sequential decision-making through rewards	Requires simulated or interactive environments; data-intensive	Optimizing TCM formulations and dosage combinations to achieve the best therapeutic effects	High, cost grows with exploration episodes; GPU recommended

SVM is a classic ML algorithm that is good at solving classification and regression problems, and is especially suitable for processing small samples and high-dimensional data. Its core idea is to accurately separate data points of different categories by constructing a hyperplane that maximizes the classification boundary, and introduces kernel functions (such as linear kernels and Gaussian kernels) to deal with nonlinear problems [28]. In AI screening of TCM, SVM can be used to predict active ingredients based on chemical fingerprints or molecular descriptors. For example, TCM compounds can be classified according to their potential efficacy in screening candidate molecules with specific functions [48], significantly improving screening efficiency and providing a reliable basis for subsequent experiments (Fig. 2A). Some researchers have used machine learning to predict hepatotoxic compounds in Polygonum multiflorum. Researchers collected a labeled dataset of drug-induced liver injury containing 2384 compounds and randomly divided it into training and test sets. They also developed a two-parameter optimization method based on SVM to improve prediction accuracy. The optimized SVM model achieved an accuracy of 0.761 and a recall of 0.834 on the test dataset. Furthermore, K-means clustering was also used to predict hepatotoxic compounds. The use of clustering algorithms to classify compounds with high hepatotoxic risk demonstrates that ML methods provide a powerful and effective tool for screening hepatotoxic substances [49]. Another study introduced the SVM pharmacodynamic prediction model to screen and predict the active ingredients in the TCM formula Naodesheng. The accuracy of the model was verified by strict jackknife tests and independent data sets. The results showed that the SVM model had lower errors and higher correlation coefficients. Therefore, it was proved that the SVM model can well grasp the relationship between chromatographic peak area and pharmacodynamic effect, and can be used to predict the efficacy of TCM compound preparations [50].

RF is a ML method based on decision trees. It constructs multiple decision trees and combines their results to make predictions. It is efficient, robust, and resistant to overfitting [26]. In TCM component screening, random forests can quickly screen out core active ingredients with potential efficacy by analyzing the chemical fingerprints or molecular descriptors of TCM compounds [48, 51, 52] (Fig. 2B). For example, by training models to predict the pharmacological activity or toxicity of compounds, random forests can handle complex nonlinear relationships while maintaining high accuracy and interpretability, providing a flexible and efficient tool for TCM screening [53]. To explore the pharmacological mechanism of the classic TCM Xiaoxu Ming Tang in the treatment of stroke, researchers designed different Bayesian ML models based on AB, kNN, CT, RF, and molecular fingerprint description algorithms. By comparison, it was found that the Bayesian model combined with the RF algorithm had better performance and could predict compounds with potential neuroprotective effects in two phenotypes [54].

DT is a tree-based ML algorithm that excels at handling classification and regression tasks. By recursively dividing the dataset, it decomposes the complex decision-making process into a series of simple conditional judgments, forming a clear decision path from the root node to the leaf nodes [24]. In TCM ingredient screening, the decision tree can screen candidate molecules related to the target efficacy layer by layer based on the structural features or physicochemical properties of TCM compounds (Fig. 2C). Its intuitive decision rules and interpretability help to discover the relationship between TCM compounds and efficacy, and provide a scientific basis for pharmacological research and ingredient optimization. Xu et al. combined a large amount of literature and high-quality data to collect 426 pain-related herbs and their targets, and used machine learning methods to identify three categories of herbs, targeting specific symptoms, targets and enriched pathways of chronic cough neuralgia, reproductive and autoimmune-related pain, and cancer pain [55].

DL automatically extracts high-level features from large-scale TCM data through multi-layer neural networks, providing a new solution for complex ingredient screening. CNN is a DL model that is good at processing grid-structured data such as images. CNN has the characteristics of local perception, parameter sharing, and hierarchical feature extraction, and performs well in image classification, target detection and other fields [40]. In the screening of TCM ingredients, CNN can be used for microscopic image analysis, such as identifying the microstructure or chemical characteristics of TCM materials [56], assisting in the quality assessment of medicinal materials and ingredient screening, providing strong technical support for TCM research (Fig. 2D). In one study, researchers collected 10,053 herbal ingredients from the Traditional Chinese Medicine Information Database (TCMID) and integrated these ingredients with seven meridian information (liver, lung, spleen, stomach, kidney, heart, and large intestine). Then, using the feature matrix determined at the herbal and compound levels, a machine learning framework was developed to predict the meridians of herbs and their component compounds. After testing multiple ML methods, it was found that herbal compounds can correspond well to meridians. This result may better help explore the research mechanism of TCM and improve its effectiveness [57]. Li et al. developed the FordNet model, which is a Chinese medicine intelligent recommendation system that integrates phenotypic and molecular information. The model uses the CNN method to extract more than 20,000 electronic diagnostic information, and then integrates it with the characteristics and molecular information of TCM prescriptions through the network to effectively select better prescription combinations. The experimental results show that FordNet performs significantly better than baseline methods, hit ratio of top 10 improved by 46.9% compared with the best baseline random forest method [58]. There are also articles reporting that the team used AI to assist in screening the efficacy and mechanism interpretation of TCM ingredients and derivatives. The research team used deep learning technology to find that berberine derivatives used in TCM for treating gastrointestinal diseases have significant antibacterial activity against multidrug-resistant Helicobacter pylori. These derivatives showed good safety and pharmacokinetic properties in vitro and in vivo experiments, providing a potential new therapy for the treatment of Helicobacter pylori infection [59]. By combining the bidirectional long short-term memory network (Bi-LSTM) with the CNN, the quantitative information of TCM in the Chinese Pharmacopoeia was analyzed and the characteristics of the prescriptions were classified based on the active ingredients. This model significantly improved the efficiency and accuracy of the prescription function classification, providing stronger technical support for the analysis of TCM big data [60].

RNN is a DL model that specializes in processing sequence data, and is able to capture the time-series relationships of the data through the recurrent connections of the hidden layers. It is particularly suitable for processing dynamic information, such as time series and biological signals, etc. [38]. In TCM ingredient screening, RNN can be used to analyze the dynamic process of TCM action, such as the time-dependent relationship between the metabolism of drug components in vivo and their action targets. By mining the dynamic features of TCM compounds, RNN helps predict the pharmacological activities of the components and optimize the pharmacodynamic mechanisms, providing a data-driven solution for studying complex TCM systems (Fig. 2E). Pi et al. collected the main anti-cancer herbs, related cancer types, meridian affiliations and their chemical information, and they used molecular graph representation in the characterization stage to replace the traditional fixed molecular fingerprint method. A new computing architecture combining GCN and RNN, GRMC, was developed for meridian sequence prediction at the level of TCM compounds [61]. This paper proposes an intelligent model based on RNN AttentiveHerb, which simulates the doctor's drug inquiry process and the prescription generation of multiple herbs. The model uses RNN to capture the dynamic association between symptoms and herbs and learns interaction patterns from traditional herbal clinical records. Two attention mechanisms are also introduced in the model, which distinguish the main symptoms on the one hand and adjust the degree of attention to different symptoms on the other hand, so as to achieve accurate simulation of complex symptoms and prescription relationships [62]. The Meta-DEP model established by Luo et al. uses Metapath2vec to generate node features by finding the shortest drug-disease path in a heterogeneous network, and combines RNN to capture sequence dependencies. The dual attention mechanism (path and node attention) aggregates node embedding, and the multi-task learning strategy enhances the prediction ability. The model combines transcriptome data and provides an innovative tool for discovering of active ingredients in TCM [63].

t-SNE is a nonlinear dimensionality reduction technique that converts high-dimensional similarities into conditional probabilities and optimally maps them onto a low-dimensional space, preserving local structure. It is particularly effective for visualizing complex data by clustering similar objects close together in 2D or 3D space [44]. When t-SNE has been utilized to visualize high-dimensional molecular descriptor data, it enables intuitive understanding of structural diversity and activity patterns among TCM compounds [43]. Visualization tools with t-SNE can facilitate hypothesis generation and help identify chemotypes with potential pharmacological relevance.

In specific tasks, the model inputs are typically chemical descriptors, such as SMILES strings, molecular fingerprints (e.g., ECFP, MACCS), and molecular graphs, which represent the compound's structure. These descriptors provide the model with a formalized understanding of the chemical properties of each compound [64]. The molecular graphs models atoms as nodes and bonds as edges, providing a more comprehensive view of the molecular structure and capturing essential topological information that is crucial for the multi-target interactions common in TCM compounds. This approach is particularly important because it enables AI models such as graph neural networks (GNNs) to analyze the structural complexity of TCM components more effectively than traditional methods. The model output depends on the specific R&D task. In drug-target interaction studies, the model output might be the predicted binding affinity between the compound and the target protein. In toxicity prediction, the output might be the classification of a compound as safe or potentially toxic [65]. In some cases, the output might also be a predicted pharmacological effect, such as modulation of tissue repair, based on the molecular features input to the model. These outputs are crucial for guiding experimental validation and optimizing the selection of drug candidates for further testing [66].

While both classic ML and DL offer valuable tools for screening active ingredients in TCM, their suitability depends on the nature of the dataset and task. ML techniques such as SVM, RF, and DT are ideal for structured, small-to-medium-sized datasets with clearly defined features like chemical descriptors or fingerprints, offering high interpretability and fast training. These models are especially useful for tasks like compound classification, toxicity prediction, or efficacy screening when data volume is limited [67]. One representative study focused on Xiaoxuming Decoction (XXMD) is a classical prescription used for stroke treatment. Researchers constructed multiple classification models—including random forest, k-nearest neighbors, and AdaBoost—and combined their outputs using a stacked naïve Bayesian approach. Virtual screening using these models identified ten candidate compounds with potential therapeutic efficacy, two of which were later confirmed through cell-based assays to protect neurons from chemically induced oxidative stress [54]. In contrast, DL models like CNNs and RNNs excel in handling large-scale, unstructured, or sequential data, such as images, time-series pharmacokinetic data, or electronic medical records, where automated feature extraction and modeling of complex relationships are needed [68]. This study developed a two-stage virtual screening framework integrating machine learning and deep learning, identifying three potential GSK-3β inhibitors from 25,000 compounds derived from TCM. This provides candidate drugs with blood–brain barrier permeability and low neurotoxicity for the treatment of Alzheimer's disease (AD). The study combined interpretable RF modeling (SHAP analysis) with KarmaDock deep learning to address the lack of interpretability in traditional "black box models," significantly improving screening accuracy [69]. In TCM research and development, model selection is not arbitrary but highly dependent on data properties, task objectives, and interpretability requirements. Researchers can follow these principles for selection: For small-scale, structured data (such as chemical component descriptors), traditional ML models (SVM, RF, DT) are more advantageous due to their resistance to overfitting and rapid training; while for large-scale, unstructured data (such as medicinal material images and medical texts), DL models (CNN, RNN) perform better due to their automatic feature extraction capabilities. Regarding task type, classification tasks are suitable for SVM, RF, and CNN; regression tasks (such as activity intensity prediction) are best suited for linear regression or SVR; and sequence prediction (such as pharmacokinetic analysis) requires time-series models such as RNN or LSTM. Furthermore, if clear decision rules are needed to guide experiments (such as key component mining), highly interpretable decision trees or logistic regression are ideal choices; and when prediction accuracy is prioritized, deep learning or ensemble learning models (such as CNN, RF) can provide superior performance. This selection framework helps optimize model strategies for specific research scenarios, balancing efficiency, accuracy, and interpretability. When choosing an AI model, it's important to consider not just data size and task complexity, but also the model's interpretability and training efficiency. These factors help ensure the model can effectively solve real-world problems in TCM, supporting its modernization.

In the development of TCM, the application of AI faces risks such as overfitting and data quality. Overfitting occurs when a model overfits the training data and fails to generalize to new data, especially when dealing with small or noisy datasets [70]. To reduce the risk of overfitting, cross-validation can be used to split the data and train the model multiple times to ensure its performance on unknown data. In addition, careful data preprocessing, such as noise filtering and data enhancement, can improve data quality and reduce the possibility of overfitting. Data quality issues directly affect model performance. In the research and development of TCM, data consistency and accuracy are crucial, but often there are inconsistencies or inaccuracies in data from different sources [71]. For example, the descriptions of the components and efficacy of certain TCM may differ in different studies, which makes ensuring data standardization and consistency a key to the successful application of AI models. Therefore, improving the data collection, standardization, and cleaning process is an effective way to improve model reliability [72]. In addition, interdisciplinary collaboration is also crucial. Combining research results from fields such as bioinformatics, cheminformatics, and pharmacology can improve data quality and accuracy at a deeper level [73]. Data cleaning technology can effectively remove erroneous or irrelevant data, fill in missing values, and ensure the comparability of data from different sources through standardization, thereby improving data uniformity. Ultimately, the training of AI models will rely on standardized datasets to ensure that they can truly reflect pharmacological effects [1]. By comprehensively considering data collection, standardization, cleaning, and interdisciplinary collaboration, a more solid data foundation can be provided for the application of AI in the field of TCM, thereby promoting the innovative development of TCM.

#### The role of AI in drug design and optimization

TCMs, as representative herbal formulations, often contain multiple bioactive ingredients that produce synergistic effects in a personalized medicine approach aiming to maximize therapeutic efficacy and minimize side effects [74]. Nowadays, AI technology has once again demonstrated its powerful potential. By integrating big data, ML and molecular simulation, AI can rapidly identify and design candidate compounds with higher bioactivity and better pharmacokinetic properties by virtually screening and optimizing the chemical structures of the active ingredients; predicting the potential efficacy and indications of TCM formulas to improve; and simulating the multiple components' synergistic or antagonistic effects among multiple ingredients, providing a scientific basis for the formulation of personalized therapeutic dosages. Through AI, the time and cost of experimental determination can be saved, the initial screening time can be shortened, and the bioactive ingredients in herbal formulas and their mechanisms of action can be more effectively understood, which may provide important insights for the rational design of multi-drug combinations for complex diseases [75, 76].

In one study, 194 TCM formulas with good efficacy in the treatment of gastric cancer were collected and, found that TCM with spleen-strengthening and qi-invigorating as the mainstay is a promising auxiliary method for the treatment of gastric cancer. Through screening, the optimal prescription composed Atractylodes macrocephala, Astragalus membranaceus, Pinellia ternata, Tangerine peel, Hedyotis diffusa and Crataegus pinnatifida. 74 active compounds and 2,128 predicted targets of the prescription were extracted from the public database. Finally, 135 genes related to gastric cancer were identified as potential targets through ML. A compound-target network was constructed, showing that quercetin, kaempferol, baicalin and nobiletin were the key active substances in the prescription. Further in vivo and in vitro experimental verification showed that the optimal prescription significantly inhibited the survival rate of gastric cancer cells and inhibited tumor progression by regulating the hTERT/MDM2-p53 signaling pathway. ML has or has the potential to guide clinical prescriptions [77]. AI-assisted drug design in the context of TCM has expanded beyond empirical pattern recognition and now enables the de novo generation and optimization of molecular scaffolds derived from herbal compounds. For instance, active compounds like quercetin and baicalin can serve as templates in AI-driven molecular optimization to improve solubility, bioavailability, and target binding affinity. These advancements greatly accelerate the lead compound discovery process and enable multitarget drug development for complex diseases such as cancer, COVID-19, and metabolic syndromes [78]. At the same time, a growing body of review literature summarizes the state-of-the-art AI tools applied in virtual screening, molecular generation, and pharmacological modeling. For instance, some researchers provided an in-depth discussion on transformer-based models and their application in compound generation and drug-likeness filtering [79], while others reviewed AI-assisted chemical profiling technologies applicable to TCM quality control [80]. Zeng and Jia further demonstrated how graph-based AI can simulate herb–herb interactions to decode compatibility principles in TCM formulations [81]. An experiment combined the Qingfei Paidu Decoction (QFPD), which can effectively protect the body from the damage of COVID-19 through multiple mechanisms of antiviral, anti-inflammatory and metabolic programming, with AI technology and a functional analysis method. It was found that 9 ingredients of QFPD showed good molecular docking scores with 2019-nCoV [82]. Some scholars have also constructed databases by organizing modern TCM and ancient literature with information on formula names, herbs and ingredients, measurements and modes of administration, and symptoms. Based on data mining and AI training, new insights into herbal medicine grouping were found from different TCM literature [83]. Another study compared the compound functional fingerprint of the Xiao Er Fu Pi (XEFP) granules (composed of six herbs: Atractylodes macrocephala, dried tangerine peel, hawthorn, Codonopsis pilosula, lotus seeds, and Poria cocos) with United States Food and Drug Administration (US FDA)-approved anti-dyspepsia FD drugs through unsupervised learning, which may help to understand the pharmacological mechanisms of TCM for the treatment of complex diseases [84]. Moreover, modern AI-assisted drug design tools allow TCM researchers to construct compound-target-pathway networks that simulate both pharmacodynamic and pharmacokinetic interactions at the system level. These approaches have led to the discovery of hybrid molecules and synthetic analogs with enhanced stability and specificity. Synthetic biology also benefits from AI algorithms in designing metabolic pathways for high-yield biosynthesis of active herbal ingredients like flavonoids and terpenoids. These efforts contribute to improving production efficiency and consistency while preserving the therapeutic essence of TCM [85].Xu et al. obtained a basic prescription for gastric cancer treatment with universal applicability through big data mining and machine learning. This model can optimize prescriptions and promote the optimization of TCM compatibility [86]. In addition, a multi-convolutional neural (MCNN) model that predicts the corresponding herbal formulas in the form of probability values by constructing symptom- ‘state element’-symptom maps (S_e_) and symptom- ‘evidence type’-symptom maps (T_s_) using a multilayer perceptron (MLP) has shown a significant improvement in the accuracy of the herbal formulas recommendation compared with other existing models of machine learning [87]. Another study used a GCN model to build ML-TCM, which can identify TCM prescription labels more delicately and thus optimize TCM prescriptions [88]. This study proposed a TCM prescription visualization method with multiple linked views based on two types of classic prescription data, which can better evaluate the characteristics of TCM prescriptions [89].

Although the above content shows to a certain extent that AI-driven TCM R&D may have great potential and has attracted much attention, the actual situation is often limited by technology, data volume, application scenarios, etc., and there are still many challenges. The gap between computational predictions and biological complexity is significant. AI models typically make predictions in simplified computer simulations, while the human body is an extremely complex system. For example, an AI model might predict that a certain TCM compound has a high affinity for a target, but it struggles to accurately simulate the compound's metabolism, bioavailability, and interactions with other components/drugs in the human body [90]. This disconnect between "in vitro" predictions and "in vivo" efficacy is a major reason why many AI-driven drug candidates fail in preclinical or clinical stages [91]. Some studies only provide preliminary computer predictions and lack experimental verification of candidate drugs, making subsequent clinical trials particularly important [1]. In addition, the complexity of TCM holistic treatment methods and the lack of understanding of the molecular mechanisms of drugs have increased the difficulty of applying AI. The uneven quality and inadequate management of TCM databases may lead to false positive analysis results [92]. Currently, the lack of high-quality and comprehensive TCM and compound databases limits the effective training and validation of AI models. Therefore, strengthening database quality control, unifying data standards, and building a classification system are considered important ways to overcome these limitations. In addition, not only TCM, but also AI has limitations in drug development, such as the problem of too small sample size. Each research team has limited development costs and often only uses a portion of the data to develop models. However, drug development is ultimately for the vast majority of people, so it is very likely that the developed models cannot support real drug development [93].

Looking ahead, beyond existing virtual screening and network pharmacology, the next generation of AI-driven research and development of novel TCM will place greater emphasis on creativity and systematic approaches. For example, by using generative AI to learn the chemical space of known active ingredients, it is hoped that novel molecular skeletons with better drug-like properties (such as higher solubility and lower toxicity) can be designed, thereby breaking through the structural limitations of TCM components. The application of AI in TCM faces dual challenges of regulation and ethics. At the regulatory level, the innovative results generated by AI face the difficulty of defining their attributes: should they be classified as new chemical entities requiring full verification, or as improved formulations of traditional prescriptions? This definition will directly affect their review pathways and clinical requirements. At the ethical level, there is a conflict between AI property rights and traditional knowledge protection. It is necessary to clarify the ownership of rights to results generated by AI models trained on ancient texts and to establish a reasonable benefit-sharing mechanism among developers, data contributors, and traditional knowledge holders.

### Development and application of AI in the pharmaceutical phase of TCM

#### Challenges in the pharmaceutical phase

**Fig. 3:**
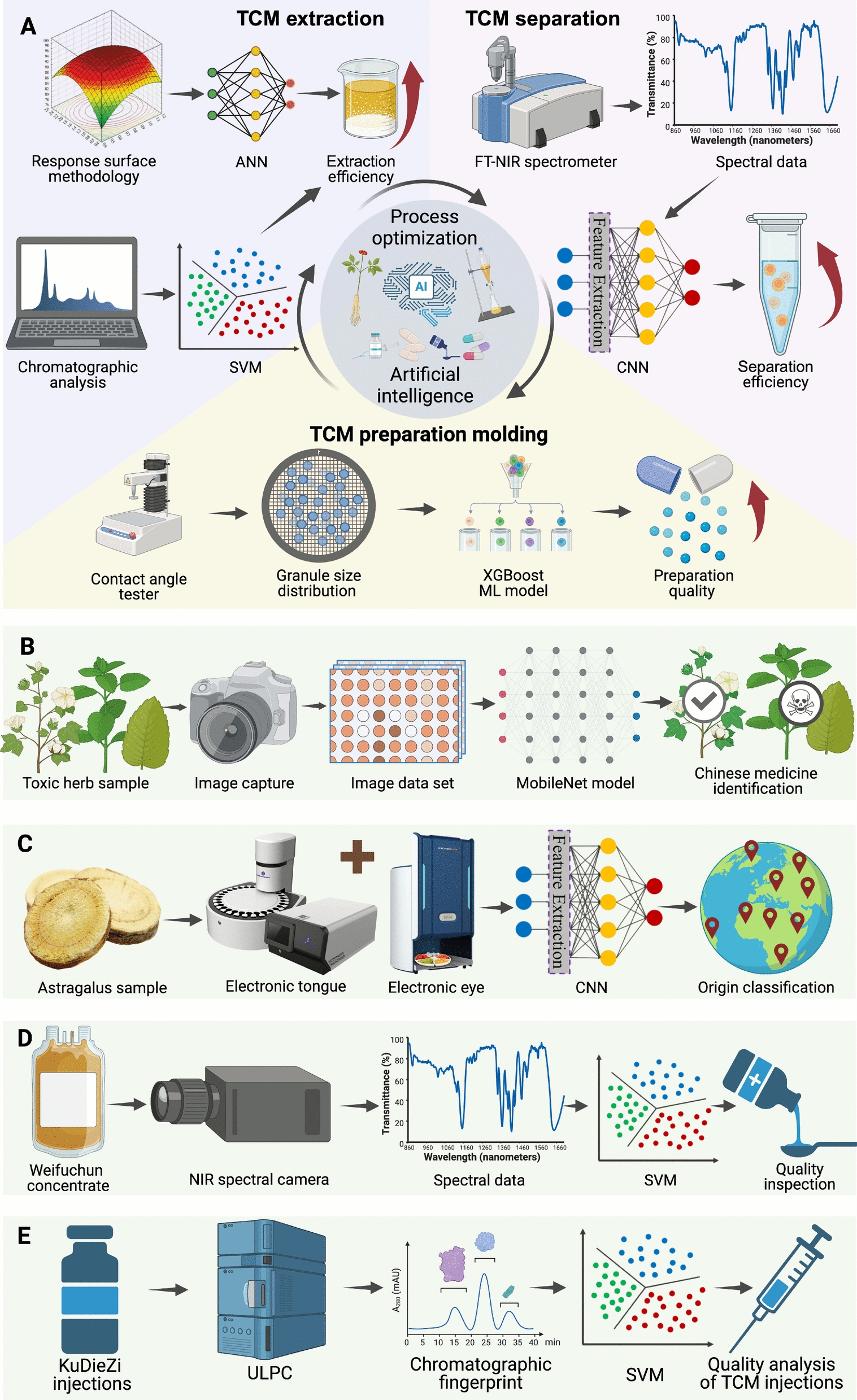
The application of artificial intelligence in the pharmaceutical phase of TCM. **A** AI technologies are used to optimize the extraction, separation, and formulation processes of TCM; **B** Combining deep learning models with computer vision for the identification and classification of TCM materials; **C** Integrating deep learning models with sensor technologies to analyze sensory data of TCM samples; **D** Utilizing machine learning models and spectroscopy for qualitative analysis, enabling quality inspection; **E** Applying machine learning models with chromatography for quantitative analysis, ensuring quality control

The pharmaceutical manufacturing stage of TCM is a complex and highly systematic process. The key links in this stage include extraction and separation, preparation and molding, and quality control and standardization [94] (Fig. 3A). In the extraction and separation stage, it is necessary to extract the effective components with biological activity from the TCM [95], while removing the ineffective components and impurities as much as possible. For example, Zhang et al. pointed out four steps (solvent extraction, distillation method, pressing and sublimation) that are generally needed to extract TCM components. At the same time, factors such as solvent selection, raw material particle size, solvent-to-solid ratio, extraction temperature and time influence the extraction efficiency [96]. The formulation and molding process aims to prepare the extracted and refined active ingredients into suitable dosage forms, to facilitate storage, transportation, and administration, as well as to meet the diversified needs of the market and clinics [97]. For example, Qu et al. explored the differences of “same formula, different dosage forms” by comparing the effects of different dosage forms on the pharmacokinetics and in vitro dissolution of analytes in the object, revealing the complexity of TCM dosage form design [98]. Quality control and standardization should be carried out throughout the entire stage of TCM manufacturing [99]. The authenticity, source, variety, purity and active ingredient content of TCM materials should be classified and tested to ensure the stability and consistency of the pharmaceutical process from the source [100]. At the same time, the active ingredient content, impurity level, stability and safety of TCM products need to be comprehensively monitored to ensure that they meet the requirements of the pharmacopoeia and relevant regulations [101].

However, there are also many difficulties in the pharmaceutical stage of TCM. In the extraction process, due to the complex nonlinear relationship between variables such as extraction temperature, solvent ratio, and time, slight parameter adjustments may significantly affect the efficacy and quality. At the same time, there is a lack of effective prediction and monitoring methods [102]. For example, researchers have found that when using microwave-assisted extraction technology to extract Strychnos potatorum polysaccharides, it was found that the polysaccharide yield (g/g), carbohydrate content (%w/w), and carbon content (%w/w) all increased steadily with the extension of extraction time, but the protein content decreased significantly with the extension of time. At the same time, microwave power also had a significant effect on the extraction yield [103]. Qiu et al. extracted Atractylodes macrocephala polysaccharides and they found that because extraction is a complex dynamic process, the concentration of active ingredients at different times is difficult to calculate [104]. In terms of quality control, due to the wide range of sources of TCM, natural factors such as the climate, soil conditions, and harvest time of the planting area, as well as subsequent processing methods, storage conditions, and other human factors, will affect the chemical composition and efficacy characteristics of the medicinal materials [105]. At the same time, in national-level drug quality standard documents such as the Chinese Pharmacopoeia and the European Pharmacopoeia, specific requirements are given regarding the source, identification, testing and analysis of TCM. These requirements are strict and wide-ranging, so there is an urgent need to optimize and adjust the quality control methods of TCM [106–108]. For example, when Yang et al. conducted a study on the quality of Atractylodes macrocephala, they found significant differences in Atractylodes macrocephala from different origins. However, the Chinese Pharmacopoeia (2020 edition) stipulates thin layer chromatography (TLC) identification and 60% ethanol extract in the quality standard of Atractylodes macrocephala, but does not specify the detection method. Although many scholars have researched on the quality control of Atractylodes macrocephala, they all require professional operations and complex analytical processes [109]. In addition, traditional analytical methods usually focus on a single or limited number of biologically active or characteristic compounds, ignoring other unknown or minor components that may play a key role in the efficacy of the drug, making it difficult to fully reflect the overall quality of the drug [110].

In such a context, the introduction of AI technology brings new hope for solving the above problems (Fig. 3). ML and DL can help optimize the effects of different parameter combinations on extraction efficiency and medicinal efficacy, thus helping researchers and developers to quickly determine the best process conditions [111]. Image recognition models can be trained by learning algorithms to quickly classify and identify TCM types and qualities [112]. AI techniques have been widely used to collect data from TCM chemical composition analysis and annotation datasets, and data post-processing tools for extracting information related to the structure of mass spectra have been heavily developed [113]. Chemical data from different technologies such as mass spectrometry, nuclear magnetic resonance spectroscopy, and chromatography can also be deeply integrated [114]. By using DL technology, multi-type classification of phytochemical components in complex matrices can be performed, thereby quickly identifying drug quality defects [115].

#### Application of AI in pharmaceutical process optimization

When optimizing the pharmaceutical process of TCM, there are many factors that influence its opertation, so there are great difficulties in its actual operation. Traditional optimization method experiments are not only costly and inefficient, but also seriously restrict the scale and standardization of the TCM pharmaceutical industry. In recent years, AI technology, especially ML technology, has played an important role in optimizing process parameters and predicting key factors in the production process. Through these technologies, unnecessary experimental links can be reduced, thereby greatly improving production efficiency. Promote the development of TCM pharmaceuticals towards intelligence, refinement and efficiency.

The extraction process of TCM usually lasts for a long time and uses fixed process parameters. Therefore, when extracting bioactive natural products from TCM, the extraction method, the nature of the extraction solvent, the particle size of the raw materials, the ratio of solvent to solid, and the extraction temperature will affect the extraction efficiency [116]. AI technology can help reveal the relationship between yield and influencing factors, predict the yield of target components under different extraction conditions, and then optimize the extraction process parameters [104]. For example, when using the maceration method [117] to extract the effective components of Baiji Wuweizi Granule (BWG), a nonlinear fitting regression equation of observations and influencing factors was first established using the radial basis function neural network (RBFNN) ML model. Subsequently, the genetic algorithm NSGA-II was used to iteratively optimize the data model and obtain multiple solutions. Finally, the combinations were ranked by the decision algorithm, and the combination ranked first was considered to be the most ideal extraction process. The effectiveness of AI-assisted extraction process optimization was demonstrated based on the results of UHPLC identification [118]. Other studies have used the Least squares support vector machine (LS-SVM) model to simulate and optimize the extraction conditions when using vacuum assisted heat reflux extraction (VAHRE) technology [119] to extract indigo. Compared with the traditional Quadratic polynomial model, the LS-SVM model showed more accurate prediction ability [120]. Chen et al. used the alkali-assisted extraction (AAE) [121] method to extract Bletilla striata polysaccharides, the main active ingredient in Bletilla striata, and used the genetic algorithm-artificial neural networks (GA-ANN) model to supplement and optimize the response surface methodology (RSM) to improve the accuracy of fitting experimental responses, prediction and biochemical process modeling. The best results were produced by swapping by two-point crossover and random mutation. The results showed that the extraction conditions obtained using the GA-ANN model significantly improved the extraction rate [122]. In addition, the ANN model and the GA-ANN model were also applied to optimize the ultrasound-assisted extraction (UAE) method and percolation [123–125].

Different extraction times are likely to lead to changes in product quality. Stopping the extraction too early may result in incomplete dissolution of the active ingredients, and over-extraction may also lead to the precipitation of harmful substances [126]. The use of AI technology can assist in analyzing the concentration changes of the target ingredients and help accurately determine the extraction endpoint, thereby improving the extraction efficiency. For example, this article used percolation [127] to extract sinomenine from Caulis Sinomenii. The spectral and concentration data of 156 percolation samples from five batches were collected. The CNN model was used to capture the relationship between the spectral data and the concentration to predict the end point of percolation. Different loss functions will affect the prediction effect of the CNN model [128]. RU et al. used the heating reflux [129] extraction method to jointly extract the active ingredients from Salvia miltiorrhiza and Chuanxiong. During the extraction, an optical measurement device was used to detect the sample online and obtain the Raman spectrum. The CNN model was used to accept the pre-processed spectral data. After comparison, in most cases, the deep learning model was much more accurate than the linear model. The concentration of the analyte can be estimated in a timely and accurate manner, providing a reference for the operator's decision-making [130].

The main purpose of TCM separation is to separate specific active ingredients from TCM extracts for further research, development and application. Chromatography, especially column chromatography, is the main method for obtaining pure natural products from complex mixtures [131]. However, the property changes between batches of plant raw materials will lead to column chromatography load concentration changes. At the same time, the resin in the chromatographic column is usually recycled without replacement, and the capacity of the resin is also decreasing. By using AI technology, the real-time detected spectral data can be analyzed and predicted to determine the separation endpoint, which can improve the separation efficiency while ensuring the consistency of the separated products. For example, when Xu et al. produced Panax notoginseng total saponins, they introduced a CNN model to analyze and predict the spectral data detected during the separation process and optimized the hyperparameters of the convolution layer. The optimized model can effectively assist operators in determining whether to stop the separation, effectively improving the operation accuracy and extraction efficiency [132]. Membrane is a functional material with selective separation [133]. Currently, membrane technology has been widely used in the separation of TCM, but membrane fouling is the biggest obstacle to the use of membrane technology in the TCM industry. Therefore, Liu et al. suggested introducing artificial neural networks into the membrane separation process of TCM to monitor and predict membrane fouling, membrane operation and permeability, and to assist researchers in making judgments [134].

The main purpose of TCM preparation molding is to process the extracted active ingredients into dosage forms that are easy to store, transport and take, while ensuring the stability of drug efficacy and bioavailability. In actual production, multiple factors such as key material dosage, properties and processing parameters will affect product quality. Through artificial intelligence technology, production parameters can be simulated and predicted to achieve optimal production quality [135]. For example, oral solid dosage forms are commonly used to process dosage forms for liquid herbal extracts. High shear wet granulation is a key process for producing oral solid dosage forms. Different wettability will affect the granule size distribution (GSD), affecting particle quality. The ML model can be used to predict the GSD of sticky powders made from Rhodiola rosea extract powder to guide the amount of binder to obtain uniform preparation particles [136]. This study addresses the challenge of digitizing quality data in multi-unit production of TCM using small sample sizes. It developed an Intelligent Quality Prediction and Diagnosis framework, which proposes a novel path-enhanced dual-integration quality prediction model to detect and correct 59 physicochemical indicators across four key manufacturing processes: honey refining, mixing, granulation, and pill making [137].

#### Application of AI in quality control and standardization

Quality control and standardization of TCM are the basis for ensuring the safety of patients' medication. In official documents on national drug standards such as the Chinese Pharmacopoeia and the European Pharmacopoeia, comprehensive and detailed quality control requirements are put forward for the entire process of TCM R&D, production, storage and use. However, traditional quality control methods require high equipment investment and professional operating skills, and often ignore the integrity of TCM and the interaction of multiple components. By introducing AI technology, combining computer vision technology, sensor technology, and chromatography and spectroscopy technology, the dependence on professional talents and expensive equipment can be significantly reduced, and the efficiency and accuracy of quality control can be improved [114, 138].

Computer vision technology can obtain fine images of medicinal materials with the help of high-definition cameras or high-resolution scanners, thereby capturing subtle appearance features. AI algorithms can automatically extract features from images and perform in-depth pattern recognition, significantly improving detection accuracy. For example, Zhang et al. collected a detailed image dataset of 1,859 angelica samples from eight regions, and used a CNN model combined with computer vision to identify the origin of angelica (Fig. 3B). Experiments have shown that this method has high accuracy [139]. In addition, Xue et al. used an improved CNN model to detect defective red ginseng based on X-ray images [140]. When identifying poisonous medicinal materials, researchers used deep learning models VGGNet16, ResNet50, and MobileNet to identify photos in the dataset. The recognition accuracy of the three models was 93.9%, 92.2%, and 95.6%, respectively. The study showed the possibility of using deep learning technology to identify various herbs [141].

Sensor technologies such as electronic noses and tongues can capture characteristics such as the smell and taste of TCM samples and generate a large amount of sensory data (Fig. 3C). However, these data are usually complex and difficult to interpret. AI technology, especially machine learning and DL algorithms, can perform an in-depth analysis of these high-dimensional data and discover the underlying patterns. For example, Jin et al. used a multimodal fusion of taste fingerprints and appearance images obtained by an electronic tongue (ET) and an electronic eye (EE) to comprehensively represent the information of Astragalus when identifying the origin and quality of Astragalus. At the same time, a lightweight hybrid model of DL algorithms that combines the advantages of CNN and Transformer was proposed. The model has good capture of both global and local features, and the method has high accuracy (99.1%) and recall rate (99.1%) [142].

Spectroscopic technology provides detailed information about the composition of Chinese medicinal materials by analyzing the absorption, reflection, and scattering characteristics of Chinese medicinal materials. Combined with AI, it can further improve data analysis capabilities and provide more accurate quality assessment and identification. For example, Li et al. used the partial least squares-discriminant analysis (PLS-DA) model to analyze and predict spectral feature data, and then identified adulterated medicinal Arnebia Radix [143]. The PLS-DA model was also used to classify hyperspectral imaging data and to distinguish the authenticity of Atractylodes macrocephala [144]. Bai et al. used Near-infrared to detect the chemical information of hydrogen-containing groups in Angelica sinensis. After preprocessing the information, the B0-LSTM algorithm was used for classification. The LSTM algorithm can consider the global sequence characteristics of spectral data, and the BO algorithm simplifies the difficulty of the LSTM algorithm in hyperparameter adjustment. Through the above combination, the authenticity of Angelica sinensis and the identification of adulteration concentration can be achieved [145]. Hui et al. proposed a fine structure recognition and segmentation technology for licorice hyperspectral images. They used a DL U-Net neural network to divide the hyperspectral data of licorice slices into phloem, xylem and pith. They used the segmented data to train ML models (LDA, SVM, and PLS-DA) and completed the classification of licorice species [146].

Through DL and ML algorithms, AI can automatically extract and identify drug component information from spectral data to achieve fast and real-time quality detection. For example, this article introduces a fast and non-contact method for quality assessment of Weifuchun (WFC) concentrate. After removing abnormal pixels from the hyperspectral images collected from the samples, the PLS and SVM algorithms were used to quantitatively calibrate the original spectra and pre-processed spectra to improve the quality prediction accuracy of WFC concentrate and enhance the quality control level of TCM preparations [147] (Fig. 3D). When conducting a quality inspection of TCM decoctions, researchers used surface enhanced Raman scattering (SERS) spectroscopy for detection and combined data analysis methods with machine learning algorithms to classify and identify Zexie-Baizhu decoctions, ultimately achieving a classification accuracy of 97.78% [148].

Chromatography is an analytical technique commonly used to separate and quantitatively analyze various drug components. In the chromatogram, the peaks of some components may be very close, and manual analysis may result in inaccurate analysis results due to human errors. AI can accurately identify and analyze peaks in massive amounts of data, achieving more accurate quantitative analysis. TCM injections are both fast and effective, but they also have frequent adverse reactions. Chromatographic fingerprinting is a commonly used qualitative evaluation method, but this method cannot accurately identify quality deviations and can easily lead to misjudgments. By using receiver operating characteristics to optimize characteristic peaks and establishing an SVM model, using two-thirds of the data as a training set and the rest as a test set, the quality monitoring and quality classification of the classified KuDieZi TCM injection can be greatly improved [149] (Fig. 3E).

In contrast to studies that only discuss datasets or theoretical AI frameworks, recent research has successfully demonstrated practical, real-world integration of AI models with TCM quality control and efficacy validation. For instance, Gao et al. applied a multi-step AI-driven strategy to the formulation Xuefu Zhuyu Oral Liquid (XZOL), widely used in cardiovascular therapy. The study combined multi-wavelength HPLC fingerprinting with pro-angiogenesis assays in zebrafish, followed by advanced spectrum-effect modeling using GRA, PLSR, BP-ANN, and a CNN integrated with Bi-LSTM and multi-head self-attention mechanisms. This approach identified seven key bioactive compounds, such as Hydroxy safflor Yellow A and Naringin, which were then validated through molecular docking, network pharmacology, and in vivo experiments. Furthermore, the integration of a CNN-based NIRS system allowed non-destructive, real-time quality control across 72 batches, highlighting the method's industrial scalability. This case provides a benchmark example of how AI can transcend theoretical potential to deliver practical tools for standardizing, evaluating, and enhancing TCM efficacy and quality control [150].

### Development and application of AI in TCM access

#### Challenges of AI-developed TCM in the access stage

Drug access refers to the whole process of new drugs from research and development to market entry and final acceptance by patients and medical systems, involving approval and registration, pricing and reimbursement, and market promotion. Its key influencing factors include regulations and policies, the clinical value of drugs, economic evaluation, and market competition environment. The main participants include regulatory agencies, payers, patients, medical service providers, and pharmaceutical companies. Drug access varies in different countries and regions, and is gradually developing in the direction of personalization and digitalization under the influence of new trends such as precision medicine and innovative payment models. The drug access of TCM and general drug access have many similarities in the process, such as clinical trials, approval and registration, pricing and reimbursement, but there are significant differences in evaluation standards and key focuses. TCM access emphasizes the comprehensive evaluation of the source of medicinal materials, the theoretical basis of traditional medicine, the overall effect of multiple components, and their clinical efficacy. At the same time needs to solve the problems of standardization and quality consistency. General drug access pays more attention to the target mechanism of a single component, clear clinical trial data support, and internationally accepted evaluation indicators.

There are many challenges in the access of TCM drugs. For example, insufficient clinical evidence and high R&D costs. Currently, the efficacy evaluation of TCM mostly relies on traditional experience and case observation, lacking standardized clinical trial data, which leads to weak scientific evidence for drug access. Therefore, rigorous scientific research, especially randomized controlled trials (RCTs), is needed to verify the efficacy and safety of TCM [151]. At the same time, the theoretical basis of TCM is challenging to quantify. The evidence of effectiveness of innovative TCM drugs in clinical trials includes TCM theory, clinical experience and pharmacodynamic studies [152, 153]. However, TCM theory is based on traditional Chinese theory and includes concepts such as yin and yang, five elements, qi, blood and body fluids. These theories are difficult to verify through the standardized evaluation system of modern medicine, making it difficult for TCM to gain widespread recognition in the process of global drug access [154]. AI-based methods, including molecular docking, ML models, and network pharmacology, can predict the binding affinities of herbal compounds to TLR4 or its associated proteins (e.g., MD2, NF-κB), as seen with agents like baicalin, berberine, and andrographolide. However, these predictions often remain at the in silico or in vitro stage. For example, although baicalin has been shown to inhibit the TLR4/NF-κB pathway and induce apoptosis in CRC cells, such effects are rarely validated in large-scale human trials due to the complexity of standardizing herbal formulations, the difficulty of isolating synergistic components, and the lack of biomarkers specific to TCM efficacy [155]. In addition, the multi-component and multi-target characteristics of TCM make its safety assessment and risk prediction more challenging, especially in the early identification and evaluation of potential safety issues such as toxicity and liver damage [153, 156].

At present, only 7 TCM have been successfully registered in the EU, including Di'ao Xinxuekang Capsules, Fannova Joint and Muscle Pain Relief Tablets, Danshen Capsules, Isatis Granules, Yufeng Ningxin Tablets, Liujunzi Pills, and Xiaoyao Tablets [157–163]. In the United States, there are currently 10 Chinese medicine products in Phase II and Phase III clinical trials at the US FDA, but they have not yet been approved and registered [164, 165]. For example, Fufang Danshen Drops is a modern innovative compound Chinese medicine independently developed by Tasly, mainly used to prevent and treat cardiovascular diseases. Despite nearly 20 years of research, the drug has not yet been successfully approved by the US FDA. In the US market, the entry threshold for herbal medicines is high. In addition to requiring strict quality control of raw materials and standardized planting and harvesting processes, large-sample, randomized, double-masked clinical trials must also be conducted, which has become a bottleneck that is difficult for Chinese patent medicines to break through.

The huge potential of AI in drug research and development has been verified, which not only significantly shortens the research and development cycle, but also improves the efficiency of drug registration and approval. A study by Boston Consulting Group shows that AI technology can reduce the time and cost of drug development from the non-clinical trial stage by 20% to 50% [166]. For example, Insilico Medicine, headquartered in New York and Hong Kong, completed the process from exploration to non-clinical trial of a candidate drug for idiopathic pulmonary fibrosis in just 30 months [167]. This efficiency far exceeds traditional methods, and the role of AI cannot be ignored. Novartis Pharmaceuticals integrates clinical trial data from multiple sources internally, uses AI technology to evaluate trial site performance, match patients with trial opportunities, and analyze digital biomarkers generated during disease progression to optimize patient recruitment time and cost control [168]. From screening active compounds to optimizing formulas and predicting therapeutic effects, AI application optimizes TCM R&D pathways and yields more reliable outcomes [169]. In addition, a report by Deloitte pointed out that in the next five years, if the top ten global biopharmaceutical companies expand the scope of AI applications, they will create a potential value of US$5 billion to US$7 billion, among which the efficiency improvement in the R&D field is the most significant, which can bring 30% to 40% value growth to various departments [153]. This efficiency and cost advantage is not only of great significance to the development of traditional drugs, but also provides new opportunities for access to TCM. In terms of assisting clinical trial design, AI can assist researchers in designing clinical trial plans through historical data analysis, including experimental group design and control group matching. At the same time, statistical learning algorithms are used to analyze historical clinical trial data to predict the optimal sample size required for new drug clinical trials to ensure the statistical power of the study [170]. In terms of assisting clinical safety research, drug-induced liver injury (DILI) is one of the most common serious adverse reactions when taking herbal medicines, and is also the main reason for terminating clinical trials or withdrawing new drugs [171]. Traditional DILI identification mainly relies on animal experiments, clinical trials, risk factor assessments, and case reports. Chen et al. used public databases and published literature as DILI datasets, combined with 9 machine learning models and 1 deep learning model to screen out hepatotoxic compounds in TCM and Chinese medicinal materials. By introducing AI technology, the risk of liver injury in clinical combination therapy can be effectively avoided [21]. Li et al. used SVM classifiers and physiologically based pharmacokinetic modeling to estimate the acceptable daily intake of hepatotoxic compounds. Estimated daily intake is important for safety assessmenting and developing over-the-counter drugs [172]. To move forward, The evaluation of TCM can adopt a hybrid evaluation frameworks, combining AI-based screening with experimental pharmacology, real-world evidence, and adaptive clinical trial designs. For instance, efforts to validate TCM formulas like Qingfei Paidu Decoction in COVID-19 treatment have shown that government-supported multi-center observational studies can provide real-world efficacy data even when RCTs are not immediately feasible [173]. Similarly, integrating multi-omics datasets with AI predictions can better reflect the systemic and dynamic interactions typical of TCM therapies. Without these integrated approaches, the translational gap between computational predictions and clinical utility will continue to hinder the modernization and internationalization of TCM.

#### Access policies and regulations

Currently, regulatory policies for AI drug development are gradually improving. Institutions such as the China National Medical Products Administration, the US FDA, and the European Medicines Agency (EMA) are all formulating guidelines to regulate the application of AI in the entire life cycle of drug development. The FDA adopts a relatively proactive stance by encouraging AI in drug development through structured guidance and sandbox programs. In contrast, the EMA emphasizes risk-based evaluation and transparency, particularly for adaptive algorithms. The NMPA, although increasingly open to AI innovation, maintains a cautious, compliance-focused approach, often requiring retrospective validation. These discrepancies influence how quickly and flexibly AI-assisted TCM formulas can transition from computational modeling to clinical application in different regions (Table 3).

**Table 3:** Key regulatory aspects of AI in TCM R&D: comparative analysis across NMPA, FDA, and EMA

Regulatory Considerations	NMPA	FDA	EMA	Implications for TCM R&D
Regulatory focus	Focus on the expedited approval process for AI/ML devices in telemedicine and public health, such as tools for pandemic prediction [204]	Evaluates AI/ML devices through a risk-based approach. Focuses on safety, effectiveness, software validation, data quality, and clinical utility [180]	Focuses on clinical validity, the ethical use of AI, and GDPR compliance to ensure data privacy and consent [205]	TCM needs to balance safety, effectiveness, and validation, especially for multi-component formulas
AI-specific guidance	Limited AI-specific guidance, mainly focusing on telemedicine and public health applications	Emerging guidelines for AI in clinical trials, especially for diagnostics and drug safety	Emphasizes GDPR compliance, ethical AI use, and clinical validity of AI tools	TCM lacks specific AI guidance, particularly for complex formulations
Data requirement	Process involves submitting detailed reports on algorithms and their updates for evaluation, including adversarial and stress testing [206]	Requires diverse and robust datasets, with clear validation and transparency for AI tools [207]	Emphasizes clinical data quality and GDPR compliance for AI tools [208]	TCM needs to meet strict data requirements, especially for AI-driven research
Traditional medicine integration	Limited focus on integrating traditional medicine, especially in AI-related regulatory frameworks	Focuses on integrating traditional medicine with conventional therapies, with AI potentially aiding personalized treatments	Acknowledges the integration of traditional medicine but underdeveloped in AI applications	Integrating TCM with AI faces regulatory challenges
Model transparency and validation	Challenges in AI model transparency and validation	Strong emphasis on algorithm transparency and validation, especially for AI tools used in drug development	High standards for transparency and validation of AI models, particularly in clinical and regulatory applications	TCM R&D must address issues of AI model transparency, ensuring the reliability and interpretability of AI-driven results, especially in clinical decision support
Challenges	Regulatory gaps in integrating TCM with AI and conventional therapies; challenges in the transparency and validation of AI models for multi-component formulations	Faces challenges in keeping up with the rapid pace of AI innovation and ensuring consistent regulation across diverse AI/ML applications	May adopt FDA-like Pre-market Clinical Performance Standards (PCPs) with added requirements for algorithm explainability and third-party audits	TCM R&D faces challenges in data standardization, AI integration with traditional medicine, and model transparency

China National Medical Products Administration (NMPA) is responsible for the registration and approval of drugs. In 2024, the NMPA issued the "List of Typical Application Scenarios of Artificial Intelligence in Drug Regulation" [174], which clarified 15 typical application scenarios of AI in drug regulation, covering four categories: access approval, daily supervision, public service, and decision support. The application of AI technology in TCM development is currently mainly guided by the “Registration Management Measures” [175]and the “Special Provisions on TCM Registration Management” in China. Regarding drug reviews, the NMPA has issued 15 typical AI application scenarios for drug supervision. The National Administration of Traditional Chinese Medicine (NATCM) of China is responsible for formulating and implementing laws, regulations, policies and development plans in the field of TCM. In the documents issued by NATCM, tasks such as data-driven intelligent drug screening and TCM new drug development are encouraged.

The US FDA requires that all new drugs must undergo adequate clinical trials. The US FDA is open to the application of AI in drug development, especially in drug discovery, clinical trial optimization, and data analysis [176]. In May 2023, the US FDA published an discussion paper on “Use of AI and ML in the Development of Drug and Biological Products” which recognized the application potential of AI and ML in drug development [177], and also discussed the considerations of AI and ML in drug development. The US FDA also published a discussion paper on "Artificial Intelligence for Drug Development" [178]. The US FDA has currently established an AI Steering Committee (AISC) to help the US FDA evaluate and ensure the safety, effectiveness and compliance of AI/ML technologies used in the pharmaceutical field. In addition, the US FDA also leads a working group within the International Council for Harmonization (ICH) to modernize clinical trial design and conduct by incorporating advanced and appropriate data management methods so that AI can be appropriately accommodated in clinical trials [179]. Although herbal medicines are not explicitly addressed in these documents, the regulatory support for AI applications in complex drug development may provide opportunities for advancing multi-component systems such as TCM, where AI can play a vital role in analyzing synergistic effects and optimizing formulations [180]. In addition, the FDA released the “Considerations for the Use of Artificial Intelligence To Support Regulatory Decision-Making for Drug and Biological Products” [181] draft in 2025, which is aimed at pharmaceutical companies that use AI to support regulatory decisions (such as preclinical, clinical, manufacturing, and post-marketing). European countries are more cautious in regulating drugs and TCM, especially in AI applications. AI-assisted TCM research and development still needs to strictly comply with EU drug regulations [182]. Europe's strict General Data Protection Regulation puts forward privacy protection requirements for the application of AI in drug R&D. In September 2024, EMA issued the “Guiding principles on the use of large language models in regulatory science and for medicines regulatory activities” to help use large language models safely, responsibly and effectively. In addition, the transparency, explainability and verification of AI algorithms are important compliance standards in the EMA approval process [183].

Overall, although AI has shown great potential in drug research and development, it still needs to be continuously improved at the regulatory level to ensure the compliance and safety of the technology. In the preclinical testing stage, artificial intelligence models simulate biological processes to predict the performance of drugs in the human body, thereby reducing reliance on animal testing. In clinical trials, AI can be used to optimize patient recruitment, improve the scientific nature of trial design, and accelerate the data analysis process to make the results faster and more accurate. In addition, AI can also simplify regulatory processes, such as helping to prepare complex documents required by the US FDA and the EMA [184]. Finally, in drug manufacturing process, AI optimizes production, ensures consistent product quality. By integrating AI technology in these key links, drug development and marketing processes can be streamlined, significantly accelerating the delivery of much-needed therapies to patients. With the NMPA successively issuing normative documents such as the "List of Typical Application Scenarios of Artificial Intelligence in Drug Regulation" [174], the "Special Provisions on the Registration Management of Traditional Chinese Medicine" [185], and the "Technical Guidelines for the Use of Real-World Evidence in Drug Regulation", the direction of scientific, evidence-based and digital regulation of TCM has become increasingly clear. These all demonstrate that the regulation and ethical construction in the field of TCM are rapidly improving, especially in terms of quality control, registration review, real-world research, adverse reaction monitoring and full-process traceability, gradually forming a modern regulatory framework. However, the deep integration and long-term development of AI in the field of TCM will inevitably be a systemic project that emphasizes both technological and institutional innovation. Technological breakthroughs must be advanced in tandem with the restructuring of the regulatory system and the establishment of ethical guidelines. In the future, efforts should be made to formulate AI explainability standards that conform to the characteristics of TCM, establish a compliance certification system covering the entire process of data, algorithms, and applications, and promote the formation of globally collaborative ethical governance principles [186].

#### AI pharmaceutical companies

**Fig. 4:**
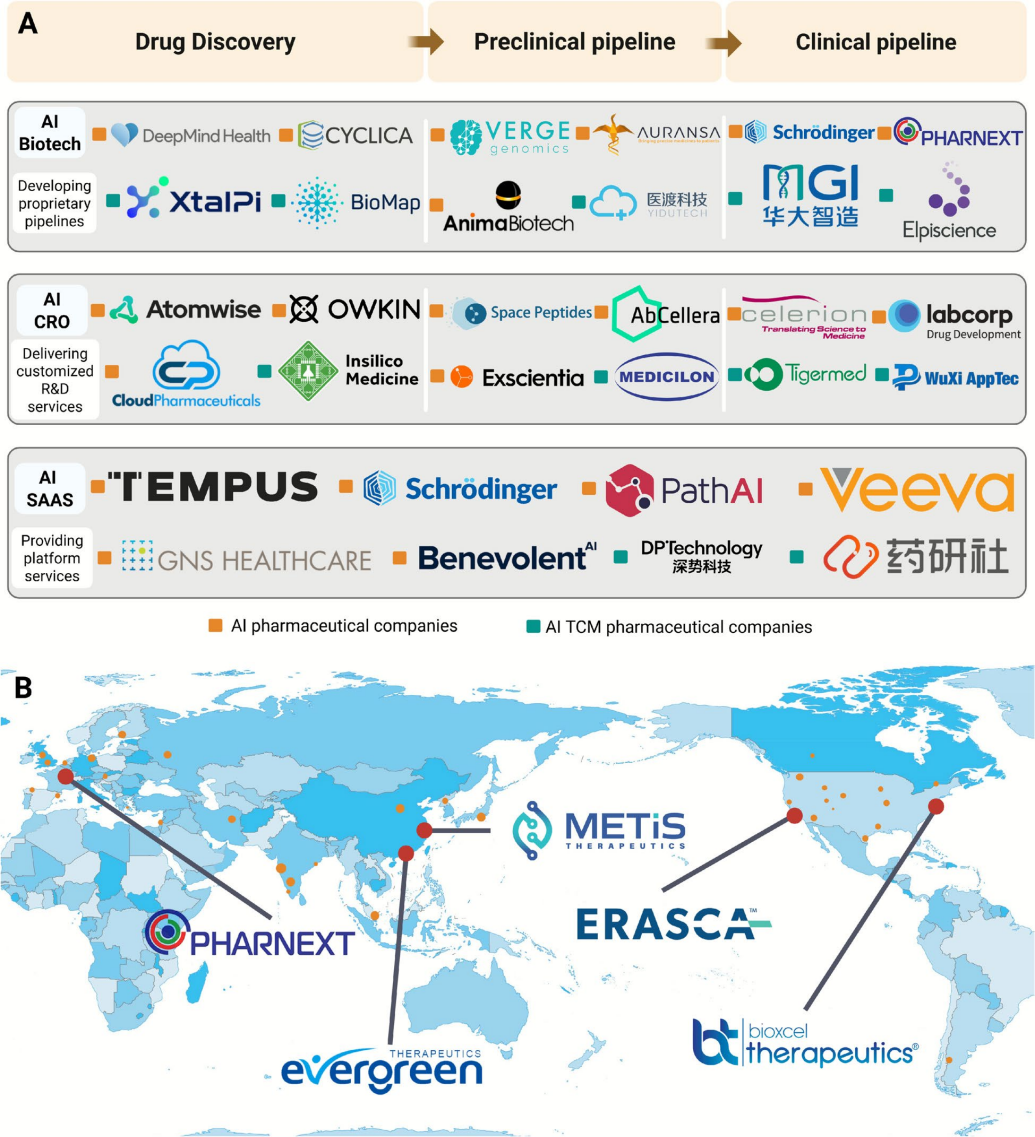
Distribution of AI Pharmaceutical Companies. **A** Some AI pharmaceutical companies and AI TCM pharmaceutical companies with AI + Biotech, AI + CRO, and AI + SaaS business models; **B** By 2023, companies entering the US FDA Phase III clinical trial stage and other companies with rapid clinical progress

Pharmaceutical companies are key participants in the drug access process. AI pharmaceutical companies are mainly divided into three categories according to their business models: AI + Biotech, AI + CRO, and Al + SaaS (software/services), Companies with various business models work together to push a large number of drugs into the clinical trial stage (Fig. 4). Among them, AI + Biotech focuses on promoting self-developed pipelines. AI + CRO companies provide customized services, participate in the development process of pharmaceutical and medical products through AI assistance, deliver phased development results to customers, or cooperate with biopharmaceutical companies to promote subsequent R&D. Al + SaaS provides AI platforms or software, indirectly providing assistance for the development of pharmaceutical and medical products, and its underlying algorithms are generally applicable (Fig. 4A).

As of 2023, 102 AI drug pipelines from 43 companies have launched clinical trials worldwide. Among the 102 AI drug pipelines, there are 5 Phase III clinical pipelines, 41 Phase II pipelines, and 56 Phase I pipelines [187]. Erasca, BioXcel, Pharnext, Jitai Pharmaceuticals, and Eglin Pharmaceuticals have made rapid progress (Fig. 4B). Erasca, a listed company in the United States, has a total of 3 AI drug clinical pipelines, of which Naporafenib was granted fast track qualification by the US FDA in 2023 for the treatment of melanoma and is in Phase III clinical trials. It uses AI technology to discover new tumor biology and innovative strategies to shut down cancer pathways. BioXcel, a listed company in the United States, has a total of 2 AI drug clinical pipelines, of which BXCL501 obtained positive data from a Phase III trial for the treatment of Alzheimer's disease in 2023, which is an artificial intelligence platform exploring old molecules and new indications [187]. Currently, Tasly and Huawei, two listed companies in China, have jointly built a “Digital Herbal Medicine” model to develop TCM AI + Biotech. The model uses nearly 3.5 million natural drug molecular data to reformulate TCM. Through the Xingdou Cloud platform, it can predict the relationship between drug targets and diseases, promote drugability evaluation and clinical transformation of basic research results, thus achieving rapid and efficient new drug development. This cooperation may promote the entry of more TCM products into clinical applications. In the TCM AI + CRO field, Shuimu Molecular and Bio-Jingfang launched ChatDD. This conversational drug development assistant serves all aspects of TCM drug development, Including early project establishment, target discovery, molecular design optimization, clinical trial design, and drug repositioning, this integration enhances the precision and accelerates the pace of R&D. Insilico Medicine's Precious3GPT (P3GPT) is an advanced AI-driven platform in the TCM AI + SaaS field, designed to bridge TCM with modern pharmacology. This multi-omics transformer model, trained on over 1.2 million omics experiments across human, mouse, and macaque tissues, analyzes aging-related molecular mechanisms. By integrating with the BATMAN-TCM2 database, which includes over 118,000 TCM compound-target interactions, P3GPT enables the identification of compounds and herbal formulas with potential geroprotective effects. The platform's AI capabilities facilitate systematic screening of TCM herbs, optimizing the design of personalized anti-aging formulations. For example, P3GPT identified 34 TCM compounds overlapping with aging-related genes in liver, lung, and muscle tissues, with the historical formula HUA SHAN WU ZI DAN containing 20 of these compounds [188]. In addition, by using AI algorithms, pharmaceutical companies can mine more potentially meaningful topics from medical data, especially the benefits and safety risk signals of different combinations of therapies after listing, thereby changing the topic selection logic of clinical research, starting from a result-oriented approach, and making efficient and full use of research resources.

At present, AI pharmaceuticals have not been verified, and industry development is facing challenges. No AI-developed drugs have been successfully launched globally, and whether AI pharmaceuticals can achieve a closed loop from target discovery to clinical trials is still under test. In 2023, at least six AI drug pipelines that have entered the clinical stage in the world have stopped research and development, and all of them have failed the critical clinical phase II. The main reasons are that the drug’s effectiveness has not been confirmed, and the clinical data is not as expected. Faced with the huge challenges of AI pharmaceutical development, many AI pharmaceutical companies have begun to merge and reorganize. In August 2024, two leading AI pharmaceutical companies, Recursion and Exscientia, announced that they had reached a final agreement to merge. Recursion will acquire Exscientia in an all-stock transaction for US$688 million. Both companies have failed to meet their expectations of AI-developed drugs [189].

## Conclusion

Throughout TCM R&D, encompassing active compound screening, formula optimization, and therapeutic effect prediction, AI integration accelerates workflows and improves decision accuracy. The achievements reviewed in this article, from AI-assisted screening of Helicobacter pylori inhibitors [59] and optimization of the neuroprotective components of Xiaoxuming Decoction [54] to the realization of quality and efficacy linkage control of Xuefu Zhuyu Oral Liquid [150], all indicate that AI has moved from the proof-of-concept stage to the practical application stage. These achievements have not only accelerated the traditional R&D process, but also provided a new perspective for the systemic regulation of complex diseases. The future development of AI in TCM will exhibit a multi-layered trend of deep integration. At the technological level, large-scale TCM models based on multimodal data fusion will become the core development direction. This involves integrating data from chemical molecules, ancient texts, and real-time physiological signals to construct intelligent systems capable of simulating the patterns of syndrome differentiation. At the clinical application level, AI will evolve from an auxiliary tool to a core component of diagnosis and treatment: in the short term, it will focus on medication safety warnings and clinical decision support; in the medium term, it will strive for personalized prescription recommendations based on phenotypic and molecular information; and in the long term, it aims to build a health management platform oriented towards system regulation. At the research paradigm level, the combination of generative AI and reinforcement learning will drive the transformation of TCM research from experience-driven to data and algorithm-driven, enabling the discovery of new molecules and the optimization of prescriptions. The realization of this development path depends on the construction of a high-quality data ecosystem and requires breakthroughs in key bottlenecks such as model interpretability, data quality control, and adaptive regulatory frameworks.

Despite this, TCM AI R&D still faces some constraints. At present, no TCM developed based on AI has been successfully launched, and the most advanced candidate drugs are only in the clinical phase II stage. This shows that although AI has shown great potential in the field of TCM, its actual transformation still faces many challenges. The authenticity and consistency of data are the main bottlenecks. Drug research in complex biological systems is easily affected by experimental conditions, resulting in uneven data quality. At the same time, the accuracy and robustness of AI modeling still need to be further improved. In addition, resource sustainability issues, such as the demand for computing resources, also limit the widespread application of AI in TCM R&D.

To address these challenges, data utilization and method design need to achieve revolutionary breakthroughs. Taking TCMBank as an example, its data inevitably relies on human judgment, so a more stringent data quality control and verification framework needs to be established. At the same time, for AI-predicted candidate drugs, the importance of experimental verification and clinical trials cannot be ignored. This is not only the key to ensuring the feasibility of R&D results but also an important link in promoting the transformation of AI-driven TCM R&D to the market. In short, AI brings far-reaching transformative potential to TCM R&D, but its realization requires overcoming current limitations and promoting collaborative innovation among disciplines. Through continuous technological progress and verification, AI is expected to open a new chapter in the field of TCM and provide strong support for developing of precision medicine.

## Data Availability

Not applicable.

## Abbreviations

AI: Artificial intelligence
TCM: Traditional Chinese medicine
R&D: Research and development
T-Aps: Pharmaceutical ingredients
LR: Linear regression
DT: Decision trees
RF: Random forest
SVM: Support vector machines
KNN: K-nearest neighbors
PCA: Principal component analysis
HMMs: Hidden Markov models
RNN: Recurrent neural network
CNN: Convolutional neural network
t-SNE: T-distributed stochastic neighbor embedding
RL: Reinforcement learning
ML: Machine learning
DL: Deep learning
TCMID: Traditional Chinese medicine information database
Bi-LSTM: Bidirectional long short-term memory network
QFPD: Qingfei Paidu Decoction
XEFP: Xiao Er Fu Pi
MCNN: Multi-convolutional neural
MLP: Multilayer perceptron
TLC: Thin layer chromatography
BWG: Baiji Wuweizi Granule
RBFNN: Radial basis function neural network
LS-SVM: Least squares support vector machine
VAHRE: Vacuum assisted heat reflux extraction
AAE: Alkali-assisted extraction
GA-ANN: Genetic algorithm-artificial neural networks
RSM: Response surface methodology
UAE: Ultrasound-assisted extraction
GSD: Granule size distribution
ET: Electronic tongue
EE: Electronic eye
PLS-DA: Partial least squares-discriminant analysis
WFC: Weifuchun concentrate
SERS: Surface enhanced Raman scattering
SDQ: Smart data query
DILI: Drug-induced liver injury
NMPA: National medical products administration
FDA: Food and drug administration
EMA: European medicines agency
NATCM: National administration of traditional Chinese medicine
AISC: AI steering committee
ISTAND: Innovative science and technology approaches for new drugs
ICH: International council for harmonization
